# Myositis-Associated Interstitial Lung Disease: The Experience of a Tertiary Center

**DOI:** 10.3390/jcm13206085

**Published:** 2024-10-12

**Authors:** Bianca Paulo Correia, Raquel Campanilho-Marques, Eduardo Dourado, Mariana Silva, Augusto Silva, Filipa Costa, Matilde Bandeira, Ana Teresa Melo, Sofia C. Barreira, João E. Fonseca

**Affiliations:** 1 Rheumatology Department, Unidade Local de Saúde Santa Maria (ULSSM), 1649-028 Lisbon, Portugal; bianca.correia@edu.ulisboa.pt (B.P.C.); raquelpcmarques@gmail.com (R.C.-M.); marianapsilva68@gmail.com (M.S.); gusto.silva.83@gmail.com (A.S.); filipamarquescosta@gmail.com (F.C.); 27219@chln.min-saude.pt (M.B.); sofi.barreira@gmail.com (S.C.B.); jecfonseca@gmail.com (J.E.F.); 2 Rheumatology Research Unit, Instituto de Medicina Molecular, Faculdade de Medicina, Universidade de Lisboa, Centro Académico de Medicina de Lisboa, 1649-028 Lisbon, Portugal; 3 Rheumatology Department, Unidade Local de Saúde Região de Aveiro (ULSRA), 3810-501 Aveiro, Portugal; 4 Aveiro Rheumatology Research Centre, Centro Académico Clínico Egas Moniz Health Alliance, 3810-501 Aveiro, Portugal; 5 Rheumatology Unit, Unidade Local de Saúde São José (ULSSJ), 1169-050 Lisbon, Portugal; anateresamelo@campus.ul.pt

**Keywords:** interstitial lung disease, idiopathic inflammatory myopathies, immunosuppressive therapy

## Abstract

**Background**: Interstitial lung disease (ILD) is a common extra-muscular manifestation of idiopathic inflammatory myopathies (IIMs), often associated with a poorer prognosis and increased mortality risk. **Methods**: This retrospective study aimed to characterize lung involvement and treatment response in an IIM cohort at a Portuguese tertiary center, followed between June 2016 and March 2024. We analyzed data from high-resolution computed tomography (HRCT) scans and pulmonary function tests (PFTs) to assess associations with autoantibody profiles and treatment regimens. **Results**: A total of 198 patients were included, with 69 (34.8%) exhibiting ILD. Antisynthetase syndrome (ASyS) and dermatomyositis were the most common diagnoses among IIM-ILD patients, with ASyS being significantly more frequent in this group than in non-ILD patients (*p* < 0.001). Anti-Jo1 and anti-MDA-5 antibodies were more frequent in ILD patients (*p* < 0.001 and *p* = 0.021), while anti-Mi2 antibodies were less common (*p* = 0.002). Non-specific interstitial pneumonia (NSIP) was the most common radiological pattern (69.5%). IIM-ILD patients presented with significantly lower forced vital capacity (FVC) and diffusing capacity of the lung for carbon monoxide (DLCO) compared to non-ILD patients (*p* < 0.001 for all values). Longitudinal analysis showed improved DLCO (*p* = 0.022) and stable or improved FVC (*p* = 0.097), especially with intravenous immunoglobulin (IVIg) and azathioprine (AZA). Combination therapies including IVIg with mycophenolate mofetil (MMF) or rituximab (RTX) also improved DLCO and FVC. Most ILD patients (89.6%) had stable HRCT patterns over time. **Conclusions**: Our findings highlight the potential for stabilizing or even improving lung function in IIM-ILD with appropriate immunosuppressive therapy, particularly with regimens incorporating IVIg and AZA, and combination therapies.

## 1. Introduction

Idiopathic inflammatory myopathies (IIMs) are a heterogeneous group of disorders in which chronic inflammation of the skeletal muscle is a common feature, leading to muscle weakness. Other organs, such as the skin, joints, lungs, gastrointestinal tract, and heart, are frequently affected in IIMs, contributing to morbidity and mortality [1].

Interstitial lung disease (ILD) is a common extra-muscular manifestation of IIM, usually associated with a poorer prognosis and an increased risk of mortality [2].

IIM patients can be grouped based on the presence of myositis-specific/myositis-associated antibodies (MSA/MAAs). Many patients with IIM have MSA/MAAs that result in distinct clinical phenotypes. Among these MSA/MAAs, anti-aminoacyl-tRNA synthetase antibodies (anti-ARS) and anti-melanoma differentiation factor 5 antibodies (anti-MDA-5) are associated with higher rates of ILD [1,3,4]. The prevalence of ILD in MDA-5+ adult patients varies widely across cohorts, ranging from 40 to 100% [5,6]. Among patients with antisynthetase syndrome (ASyS), the prevalence of ILD ranges from 70 to 95%, depending on the cohort [6,7,8]. However, ILD may also be present in patients without these autoantibodies. Therefore, all patients with IIM should be screened for lung involvement with chest high-resolution computed tomography (HRCT) and pulmonary function tests (PFTs). In fact, one-third to three-quarters of patients with IIM have evidence of ILD on HRCT scans of the chest [3,9,10,11,12,13]. The most common features of IIM-ILD in HRCT include bilateral reticulations and ground-glass opacities, favoring a non-specific interstitial pneumonitis (NSIP) pattern; ground-glass opacities and consolidations consistent with organizing pneumonia (OP); and honeycombing, reticulations, and traction bronchiectasis indicating advanced fibrosis in usual interstitial pneumonia (UIP) [4,14,15,16,17,18]. The distribution of these patterns partially depends on the IIM subtype, with NSIP predominating in ASyS and OP in MDA-5-dermatomyositis [19,20].

PFTs are frequently used to evaluate patients with respiratory symptoms, help define the severity of pulmonary disease, and assess therapeutic response. The typical pattern in IIM patients is a restrictive pattern, defined as a decrease in total lung capacity (TLC), forced vital capacity (FVC), diffusing lung capacity for carbon monoxide (DLCO), forced expiratory volume at first second (FEV1), functional residual capacity (FRC), and residual volume (RV), with an increased FEV1:FVC ratio [4].

Patients with myositis-associated ILD present unique diagnostic and therapeutic challenges that are best approached through multidisciplinary collaborations involving experienced rheumatologists, pulmonologists, and radiologists [21]. Clinical and radiologic features, pulmonary function testing, and autoantibody expression can influence treatment responsiveness and prognosis.

With this work, we aimed to characterize a large Portuguese cohort of patients from a tertiary center with IIM-associated ILD, including data from HRCT and PFTs and their association with autoantibody profiles, treatment regimens, and treatment response.

## 2. Materials and Methods

We retrospectively collected data from all IIM patients followed in the Myositis Clinic of our Rheumatology Department at Unidade Local de Saúde Santa Maria (ULSSM), Lisbon, Portugal, from June 2016 to March 2024 [22]. We included patients with a confirmed IIM diagnosis based on the EULAR/ACR 2017 classification criteria who were 18 years of age or older at the time of diagnosis. We also included patients with a diagnosis of IIM-associated/overlapping connective tissue diseases according to international criteria, including the 2016 ACR/EULAR classification criteria for primary Sjögren’s syndrome, the 2019 EULAR/ACR classification criteria for systemic lupus erythematosus (SLE), the 2013 ACR/EULAR classification criteria for systemic sclerosis, the 2010 ACR/EULAR classification criteria for rheumatoid arthritis, and Sharp’s, Kasukawa, Alarcón-Segovia, or Kahn’s diagnostic criteria for mixed connective tissue disease (MCTD).

This study was approved by the Ethics Committee of Centro Académico de Medicina de Lisboa (CAML), in accordance with the principles of the Declaration of Helsinki.

Patient demographic data, clinical manifestations, laboratory findings, chest X-rays, HRCT chest scans, and PFTs were collected from electronic medical files. Patients were classified as having ILD upon confirmation through chest HRCT. For patients with ILD, data regarding the predominant HRCT pattern at the time of ILD diagnosis, including NSIP, UIP, OP, lymphocytic interstitial pneumonia (LIP), acute interstitial pneumonitis (AIP), and desquamative interstitial pneumonia (DIP), were recorded. For all patients, with or without ILD, we recorded the first and the last available chest HRCT, with an interval of at least one year between them. Data on PFTs were collected for all patients, including the baseline and the last PFTs available, also spaced by a minimum of one year. For the response-to-treatment analysis, we selected ILD patients with a restrictive pattern at the baseline as defined by the 2005 American Thoracic Society and European Respiratory Society (ATS/ERS) guidelines (TLC below the 5th percentile of the predicted value), with an FVC ≤ 70% and/or DLCO ≤ 60%, with at least one year of follow-up and two different PFT evaluations [23].

The data were analyzed using SPSS version 29.0.2.0 (SPSS, Inc., Chicago, IL, USA). Descriptive statistics were presented as mean ± standard deviation (SD) for continuous and normal variables, as median (interquartile range, IQR) for continuous non-normal variables, and as absolute and relative frequencies for categorical variables. The Shapiro–Wilk test was used to test normal distribution for continuous data. Associations between the categorical variables were tested using the chi-square or Fisher’s exact test. Associations between categorical and continuous variables were tested using Student’s *t*-test for independent and paired samples, McNemar, or Mann–Whitney U tests, as appropriate. Definite and likely associations were defined by *p*-value < 0.050 and *p*-value < 0.100, respectively.

## 3. Results

### 3.1. Patient Clinical and Demographic Characteristics

A total of 198 patients with IIM were included, of whom 144 (72.7%) were female, with a median age at diagnosis of 47.5 (IQR 31.0) years and a median disease duration of 5.0 (IQR 7.0) years.

Sixty-nine patients (34.8%) had ILD based on HRCT scans. Of these, 48 patients (69.5%) exhibited a radiological pattern compatible with NSIP, of whom 7 (14.6%) had fibrotic NSIP. Seven patients [7/69 (10.1%)] had a UIP pattern, while the remaining patients had OP [6/69 (8.7%)], AIP [3/69 (4.3%)], OP/NSIP overlap [2/69 (2.9%)], LIP [2/69 (2.9%)], and DIP [1/69 (1.4%)].

Table 1 details the clinical, functional, and serological features of patients with ILD compared to those without ILD.

The most common diagnoses among IIM-associated ILD patients were ASyS [33/69 (47.8%)] and dermatomyositis (DM) [17/69 (24.6%)]. Ten patients [10/69 (14.5%)] had overlap syndromes [4 (5.8%) with LES, 4 (5.8%) with systemic sclerosis, 1 (1.4%) with Sjögren’s syndrome, and 1 (1.4%) with rheumatoid arthritis]. Four patients [4/69 (5.8%)] had MCTD, 3/69 (4.3%) patients had undifferentiated connective tissue disease, and 2/69 (2.9%) patients had polymyositis (PM). Comparing IIM subtypes between ILD and non-ILD groups, ASyS was significantly more common in the ILD group than in the non-ILD group [7/129 (5.4%), *p* < 0.001]. On the other hand, DM and PM were significantly less frequent in ILD patients compared with non-ILD [65/129 (50.4%), *p* < 0.001; 15/129 (11.6%), *p* = 0.037]. The remaining diagnoses were not statistically different in the ILD and non-ILD groups (Table 1).

In the group of ILD patients, 68.1% (47/69) were female, with a median age at diagnosis of 50.0 (IQR 30.0) years and a median disease duration of 5.0 (IQR 6.0) years. There were no significant differences regarding sex, age at diagnosis, or disease duration between patients with and without ILD.

Patients with ILD more commonly reported exertional dyspnea [39/62 (62.9%)], grade 1 on the mMRC (modified Medical Research Council) scale [21/39 (53.8%)] and grade 2 on the NYHA (New York Heart Association) classification [19/39 (48.7%)], asthenia [37/61 (60.7%)], and cough [34/61 (55.7%)], most commonly non-productive cough [26/61 (42.6%)]. Smoking status (current, past, or never smoking) had no statistically significant impact on symptoms during follow-up (*p* = 0.472 at baseline, *p* = 0.192 at the last visit).

### 3.2. Patient Laboratory Findings and Autoantibody Profiles

The most prevalent autoantibodies among patients with ILD were anti-Jo1 [25/64 (39.1%)] and anti-MDA-5 [8/64 (12.5%)], both significantly more prevalent than in non-ILD patients [vs. 4/106 (3.8%), *p* < 0.001; vs. 3/106 (2.8%), *p* = 0.021]. On the other hand, Mi2 antibodies were significantly less frequent than in patients without ILD [1/64 (1.6%) vs. 18/106 (17.0%), *p* = 0.002] (Table 1). In the ILD group, 22 patients (61.1%) were also positive for Ro52.

Positivity for antinuclear antibodies (ANAs) was significantly more frequent in the ILD group [52/65 (80.0%) vs. 69/114 (60.5%), *p* = 0.007].

In the ILD group, the median highest CK value was 271.0 (IQR 1155) international units per liter (IU/L) versus 343.0 (IQR 1582) IU/L in patients without ILD. The difference between the two groups was not statistically significant (*p* = 0.203).

### 3.3. Pulmonary Function Evolution and HRCT Progression

IIM-associated ILD patients presented with lower FVC (% predicted) (82.5 ± 25.4 vs. 99.2 ± 23.0, *p* < 0.001), single-breath DLCO (SB DLCO) (%) (62.5 ± 19.5 vs. 82.4 ± 16.7, *p* < 0.001), and DLCO corrected for alveolar volume (DLCO-VA) (%) (83.3 ± 15.6 vs. 97.9 ± 38.6, *p* = 0.011) values than non-ILD patients (Table 1).

**Figure 1:**
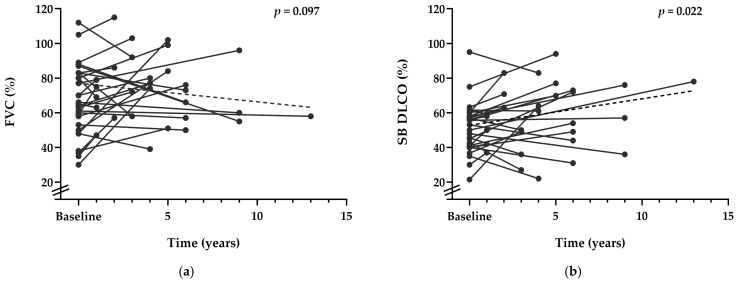
Evolution of PFTs between baseline and the last visit for each patient with ILD (N = 31). Each point represents a specific measurement, with lines connecting points for each patient to show the temporal trajectory. The dashed line represents the trend in the mean values: (**a**) evolution of FVC (% predicted value) over time; (**b**) evolution of SB DLCO (% predicted value) over time.

From the first to the last visit [mean follow-up 4.1 ± 4.0 (minimum 1.0; maximum 18.0) years], 60% of the patients experienced either an improvement or no decline in FVC (%) (*p* = 0.097), and a significant mean improvement in SB DLCO (%) was observed (*p* = 0.022) (Figure 1a,b).

When comparing HRCT changes between baseline and the most recent evaluation, the majority of ILD patients showed stability or improvement in radiologic patterns. Specifically, in 89.6% (43/48) of patients with at least two HRCT scans for comparison, there was no progression or new-onset fibrosis, no progression of ground-glass opacities [42/47 (89.3%)], honeycombing [41/46 (89.1%)], or bronchiectasis [35/46 (76.1%)].

### 3.4. Treatment Regimens and Response

Thirty-one patients with ILD were included in the response-to-treatment analysis based on the previously mentioned criteria. The baseline characteristics of these patients (N = 31) are represented in Table 2.

All patients (100%) received oral prednisolone at some point during the follow-up period. At the time of the last visit, 25/31 (80.6%) patients were receiving combination therapy, defined as treatment with at least one conventional synthetic disease-modifying antirheumatic drug (csDMARD) and/or a biologic disease-modifying antirheumatic drug (bDMARD) and/or oral prednisolone. Twenty-five patients (80.6%) received mycophenolate mofetil, 19/31 (61.3%) received hydroxychloroquine, 14/31 (45.2%) methotrexate, 12/31 (38.7%) azathioprine, 11/31 (35.5%) rituximab, 8/31 (25.8%) intravenous immunoglobulin (IVIg), 6/31 (19.4%) cyclophosphamide, 4/31 (12.9%) tacrolimus, 2/31 (6.5%) cyclosporine, 2/31 (6.5%) leflunomide, and 1/31 (3.2%) an anti-tumor necrosis factor alpha (anti-TNF α) (adalimumab). One patient received anti-fibrotic therapy with nintedanib.

**Figure 2:**
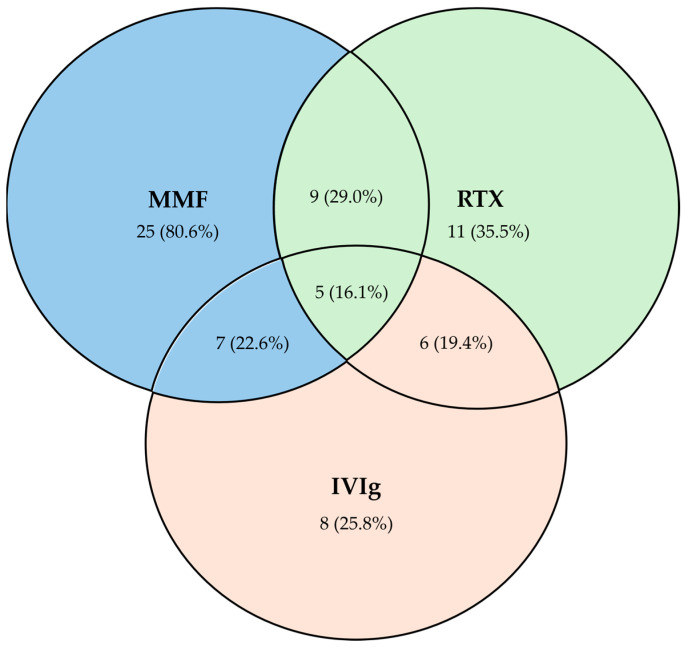
Venn diagram illustrating combinations of the most efficacious therapies used in IIM patients with interstitial lung disease followed at our center (N = 31). MMF: mycophenolate mofetil; RTX: rituximab; IVIg: intravenous immunoglobulin.

The number of IVIg cycles performed by patients ranged from 1 to 27, with a median of 6 cycles (IQR 16.0). Patients treated with IVIg showed a significant improvement in FVC (% predicted) [17.0 ± 16.8 (*p* = 0.025)] and in SB DLCO (%) values [14.2 ± 12.8 (*p* = 0.016)] (Table 3). Patients treated with azathioprine (AZA) also showed a significant improvement in SB DLCO (%) [9.1 ± 12.5 (*p* = 0.029)] and in DLCO-VA (%) values [9.4 ± 11.4 (*p* = 0.029)]. Additionally, we found a significant improvement in patients treated with a combination of mycophenolate mofetil (MMF) and IVIg [7/31 (22.6%)] and in patients treated with rituximab (RTX) and IVIg [6/31 (19.4%)] (Figure 2 and Table 3).

A likely association was observed between the stability or improvement of bronchiectasis progression and treatment with RTX (*p* = 0.063). No other significant associations were found concerning HRCT features. Of note, none of the patients were untreated.

## 4. Discussion

In our study, we found that more than a third of IIM patients in a large Portuguese cohort had ILD, based on HRCT scans, with NSIP being the most common radiological pattern (69.5%). These findings are consistent with the ILD prevalence reported in other studies, ranging from 25% to 50% [3,5,6,7,13,24]. Consistent with the existing literature, we also observed a significant predominance of ASyS and DM among IIM-associated ILD patients. The significant association of anti-Jo1 and anti-MDA-5 antibodies in our cohort is also in concordance with the established link between these autoantibodies and IIM phenotypes associated with ILD risk [1,5,6,7,25]. In contrast, the lower frequency of Mi2 antibodies in ILD patients is consistent with studies that suggest a lower ILD risk in Mi2-positive IIM patients [25].

The significantly lower FVC, SB DLCO, and DLCO-VA values in IIM-associated ILD patients at baseline, compared to those without ILD, highlight the negative impact of ILD on lung function and the need for early detection and intervention. A large multicentric study conducted in Italy and France reported similar reductions in lung function parameters in their cohort of IIM-associated ILD patients [26]. Notably, our study demonstrated that most patients (60.0%) exhibited either an improvement or no decline in FVC values, along with a clear improvement in DLCO, from the first to the last visit, suggesting a significant potential for lung function stabilization and improvement with immunosuppressive treatment. These findings are consistent with studies that highlight the effectiveness of various immunosuppressive therapies in managing IIM-associated ILD [24,27]. The observation that 89.6% of patients showed stability or improvement in radiological patterns further supports the potential of early appropriate treatment to limit disease progression, thus mitigating the risk of irreversible lung damage.

The significant improvement in FVC and SB DLCO in patients treated with IVIg is particularly remarkable in our study. Although the vast majority of evidence supporting the use of IVIg in patients with IIM is focused on skin and muscle findings, case reports and case series suggesting the usefulness of IVIg as an adjunct therapy in patients with pulmonary involvement are increasing [10,24,28,29]. Our findings suggest a potential benefit of IVIg in improving lung function in IIM-associated ILD patients. However, we acknowledge that this effect should be interpreted with caution, as all patients in our study were receiving combination therapies, including oral prednisolone and/or other csDMARDs. These findings warrant further investigation through larger, prospective studies to better understand the role of IVIg in this context. The significant improvement in DLCO values in patients treated with azathioprine is also noteworthy, as azathioprine is a commonly used csDMARD across several connective tissue diseases. This finding supports the use of azathioprine as a potential first-line treatment option for IIM-associated ILD, although further research is needed to confirm its efficacy compared to other immunomodulators [6,10,24]. The improvements in patients treated with MMF and IVIg, as well as RTX and IVIg, suggest potential synergistic effects of combination therapy [6,10,25,27]. The concept of combination therapy has been explored in several longitudinal studies and case reports, particularly in cases of MDA5-positive dermatomyositis with interstitial lung disease. Promising results have been observed with combinations involving MMF, RTX, tofacitinib, and antifibrotic agents [30,31,32]. Additionally, IVIg in combination with csDMARDs (such as methotrexate) has demonstrated promising results in anti-HMGCR myopathy [33]. This highlights the need for individualized treatment approaches tailored to the specific characteristics of each patient. We also acknowledge the potential side effects of certain therapies as a limitation of our study, as they may require dose adjustments based on patient tolerance. Gastrointestinal adverse events are common, especially with MMF, particularly when combined with other therapies such as antifibrotic agents [34]. Additionally, while the use of multiple immunosuppressive agents can be beneficial, as demonstrated by our results, it may also increase the risk of infections [35,36,37]. In this context, some patients treated with RTX receive IVIg therapy due to RTX-induced hypogammaglobulinemia [38,39].

The way PFT values were reported in our study [as percentage of predicted values rather than as percentiles or z-scores, as recommended by the most recent 2022 ERS/ATS (European Respiratory Society and American Thoracic Society) guidelines] is also a limitation of our work [40]. This limitation occurred because most tests performed at our center were reported according to the previous ATS/ERS 2005 and ATS 1991 guidelines [23,41]. Since our objective was to compare baseline tests (some from 2016 to 2017) with the most recent ones, we chose to use percentage values to standardize the results. However, some results in the treatment response analysis may be overestimated or underestimated, particularly in patients with values near the extremes of the normal limits when using % of predicted values. This limitation could be addressed in the future by adopting z-scores to report lung function tests across all centers.

No randomized controlled trials have been conducted to assess the efficacy and tolerance of corticosteroids and immunosuppressive drugs in patients with IIM-associated ILD. There are no specific guidelines for the treatment of ILD in patients with IIM. Thus, the indication for an immunosuppressive agent and the choice of the molecule are mostly based upon expert opinion, familiarity and experience of the clinician with a particular drug, and its adverse event profile. Corticosteroids remain the cornerstone and first-line treatment for IIM-associated ILD. A number of drugs have been used, including azathioprine, methotrexate, cyclophosphamide, cyclosporin, tacrolimus, rituximab, and IVIg; however, no large studies have been published on their use in this context [24,42]. We suggest conducting multicenter, prospective studies that compare different therapeutic options head-to-head and aim to unequivocally demonstrate the superiority of these therapies versus placebo (particularly in the case of new drugs) and versus alternative treatments. Ideally, these should be large-scale, randomized studies, although we acknowledge the potential challenges associated with this, given the rarity of these diseases.

Our study provides valuable insights into the characteristics, treatment regimens, and response of IIM-associated ILD in a large cohort from a Portuguese tertiary hospital. The longitudinal follow-up and detailed assessment of lung function and HRCT changes are particular strengths of this work. However, a standardized therapeutic approach to these patients is lacking, and prospective studies in the field are needed to determine optimal treatment regimens [10].

## Figures and Tables

**Table 1:** Clinical, functional, and serological features of IIM patients with and without interstitial lung disease.

Variables	ILD Patients (N = 69)	Non-ILD Patients (N = 129)	*p* Value
Age at diagnosis (in years), median (IQR)	50.0 (30.0)	45.0 (32.0)	0.341
Disease duration (in years), median (IQR)	5.0 (6.0)	5.0 (7.0)	0.650
Female, *n* (%)	47 (68.1)	97 (75.2)	0.287
Mortality, *n* (%)	5 (7.2)	10 (7.8)	0.898
**Diagnosis**			
Antisynthetase syndrome, *n* (%)	33 (47.8)	7 (5.4)	**<0.001**
Dermatomyositis, *n* (%)	17 (24.6)	65 (50.4)	**<0.001**
Overlap syndrome with polymyositis, *n* (%)	10 (14.5)	16 (12.4)	0.678
Mixed connective tissue disease, *n* (%)	4 (5.8)	13 (10.1)	0.306
Undifferentiated connective tissue disease, *n* (%)	3 (4.3)	6 (4.7)	1.000
Polymyositis, *n* (%)	2 (2.9)	15 (11.6)	**0.037**
Inclusion body myositis, *n* (%)	0 (0.0)	2 (1.6)	0.544
Immune-mediated necrotizing myopathy, *n* (%)	0 (0.0)	5 (3.9)	0.165
**Clinical features**			
Arthritis, *n*/*N* (%)	44/67 (65.7)	38/128 (29.7)	**<0.001**
Myositis, *n*/*N* (%)	45/68 (66.2)	94/128 (73.4)	0.287
Raynaud’s phenomenon, *n*/*N* (%)	35/68 (51.5)	39/128 (30.5)	**0.004**
Mechanic’s hands, *n*/*N* (%)	20/68 (29.4)	18/128 (14.1)	**0.010**
Gottron’s sign, *n*/*N* (%)	19/68 (27.9)	38/128 (29.7)	0.798
Calcinosis, *n*/*N* (%)	3/68 (4.4)	11/128 (8.6)	0.387
**Other organ involvement**			
Esophageal involvement, *n*/*N* (%)	11/68 (16.2)	7/128 (5.5)	**0.013**
Heart involvement, *n*/*N* (%)	4/69 (5.8)	5/129 (3.9)	0.722
Pulmonary hypertension, *n*/*N* (%)	5/69 (7.2)	3/129 (2.3)	0.130
**Pulmonary function tests**			
FVC % of predicted value (at baseline) (mean ± DP)	82.5 ± 25.4	99.2 ± 23.0	**<0.001**
SB DLCO % (at baseline) (mean ± DP)	62.5 ± 19.5	82.4 ± 16.7	**<0.001**
DLCO-VA % (at baseline) (mean ± DP)	83.3 ± 15.6	97.9 ± 38.6	**0.011**
**Manual Muscle Testing (MMT8 80/80)**			
Lowest MMT8, median (IQR)	77.0 (10.0)	76.0 (16.0)	0.228
Maximum	80.0	80.0	-
Minimum	42.0	10.0	-
**Modified DAS skin score (0–5)**			
Highest modified DAS skin, median (IQR)	1.0 (3.0)	1.0 (2.0)	0.223
Maximum	5.0	5.0	-
Minimum	0.0	0.0	-
**Highest CK value (IU/L), median (IQR)**	271.0 (1155)	343.0 (1582)	0.203
**Main autoantibody profiles**	**N = 64**	**N = 106**	
Anti-Jo1, *n* (%)	25 (39.1)	4 (3.8)	**<0.001**
Anti-MDA5, *n* (%)	8 (12.5)	3 (2.8)	**0.021**
Anti-PM/Scl75, *n* (%)	5 (7.8)	3 (2.8)	0.154
Anti-PL12, *n* (%)	5 (7.8)	3 (2.8)	0.154
Anti-PL7, *n* (%)	4 (6.3)	2 (1.9)	0.200
Anti-Ro52, *n* (%)	4 (6.3)	10 (9.4)	0.464
Anti-U1-RNP, *n* (%)	3 (4.7)	14 (13.2)	0.073
Anti-TIF1γ, *n* (%)	2 (3.1)	9 (8.5)	0.211
Anti-PM/Scl100, *n* (%)	1 (1.6)	5 (4.7)	0.411
Anti-Mi2, *n* (%)	1 (1.6)	18 (17.0)	**0.002**
Anti-Ku, *n* (%)	1 (1.6)	7 (6.6)	0.261
Anti-Ro60, *n* (%)	1 (1.6)	1 (0.9)	1.000
Anti-NOR90, *n* (%)	1 (1.6)	0 (0.0)	0.376
Anti-EJ, *n* (%)	1 (1.6)	0 (0.0)	0.376
Anti-SAE1, *n* (%)	1 (1.6)	6 (5.7)	0.257
Anti-Th/To, *n* (%)	1 (1.6)	1 (0.9)	1.000
Anti-SRP, *n* (%)	0 (0.0)	6 (5.7)	0.084
Anti-NXP2, *n* (%)	0 (0.0)	4 (3.8)	0.298
Anti-RNA polymerase III, *n* (%)	0 (0.0)	2 (1.9)	0.528
ANAs, *n*/*N* (%)	52/65 (80.0)	69/114 (60.5)	**0.007**

*n*: number of patients positive for the variable of interest; N: number of patients without missing information regarding the variable of interest; FVC: forced vital capacity; SB DLCO: single-breath diffusing capacity of the lung for CO; DLCO-VA: diffusing capacity of the lung for CO corrected for alveolar volume; DAS: disease activity score; CK: creatine kinase; IU/L: international units per liter; Jo1: histidyl tRNA synthetase; MDA5: melanoma differentiation-associated gene 5; PL7, PL12: anti-alanyl tRNA synthetase; PM/Scl: polymyositis/scleroderma; RNP: ribonucleoprotein; TIF1γ: transcription intermediary factor 1-gamma; NOR90: nucleolar organizer region 90; EJ: glycyl tRNA synthetase; NXP2: nuclear matrix protein 2; SAE: small ubiquitin-like modifier activating enzyme; SRP: signal recognition particle; ANAs: antinuclear antibodies. Values in bold indicate statistical significance at the 0.05 level (*p* < 0.05).

**Table 2:** Clinical, functional, and serological features of selected patients with interstitial lung disease.

Characteristics of ILD Patients	Total (N = 31)
Age at diagnosis (in years), median (IQR)	45.0 (21.0)
Disease duration (in years), median (IQR)	5.0 (5.0)
Female, *n* (%)	20 (64.5)
Mortality, *n* (%)	1 (3.2)
**Smoking status**	
Non-smoker, *n*/*N* (%)	12/24 (50.0)
Ex-smoker, *n*/*N* (%)	11/24 (45.8)
Smoker, *n*/*N* (%)	1/24 (4.2)
**Diagnosis**	
Antisynthetase syndrome, *n* (%)	16 (51.6)
Dermatomyositis, *n* (%)	7 (22.5)
Overlap syndrome with polymyositis, *n* (%)	4 (12.9)
Mixed connective tissue disease, *n* (%)	3 (9.7)
**Clinical features**	
Arthritis, *n* (%)	25 (80.6)
Myositis, *n* (%)	19 (61.3)
Raynaud’s phenomenon, *n* (%)	17 (54.8)
Mechanic’s hands, *n* (%)	9 (29.0)
Gottron’s sign, *n* (%)	7 (22.6)
Calcinosis, *n* (%)	1 (3.2)
**Other organ involvement**	
Esophageal involvement, *n* (%)	5 (16.1)
Heart involvement, *n* (%)	4 (12.9)
Pulmonary hypertension, *n* (%)	3 (9.7)
**HRCT patterns**	
Nonspecific interstitial pneumonia (NSIP), *n* (%)	19 (61.3)
Fibrosing NSIP, *n* (%)	3 (15.8)
Usual interstitial pneumonia (UIP), *n* (%)	5 (16.1)
Organizing pneumonia (OP), *n* (%)	4 (12.9)
OP/NSIP overlap, *n* (%)	1 (3.2)
Acute interstitial pneumonitis (AIP), *n* (%)	1 (3.2)
Lymphocytic interstitial pneumonia (LIP), *n* (%)	1 (3.2)
**Pulmonary function tests**	
FVC % (last–baseline) (mean ± DP) (*p* value)	6.0 ± 19.2 (0.097)
SB DLCO % (last–baseline) (mean ± DP) (*p* value)	6.2 ± 14.1 (**0.022**)
DLCO-VA % (last–baseline) (mean ± DP) (*p* value)	4.2 ± 14.5 (0.149)
**Manual Muscle Testing (MMT8 80/80)**	
Lowest MMT8, median (IQR)	76.5 (10.5)
Maximum	80.0
Minimum	56.0
**Modified DAS skin score (0–5)**	
Highest modified DAS skin, median (IQR)	0.5 (3.0)
Maximum	5.0
Minimum	0.0
**Highest CK value (IU/L), median (IQR)**	257.5 (850.0)
**Main autoantibody profiles**	**N = 30**
Anti-Jo1, *n* (%)	10 (33.3)
Anti-MDA5, *n* (%)	5 (16.7)
Anti-PL7, *n* (%)	3 (10.0)
Anti-PM/Scl75, *n* (%)	3 (10.0)
Anti-PL12, *n* (%)	2 (6.7)
Anti-Ro52, *n* (%)	1 (3.3)
Anti-Ku, *n* (%)	1 (3.3)
Anti-TIF1γ, *n* (%)	1 (3.3)
Anti-NOR90, *n* (%)	1 (3.3)
Anti-SAE1, *n* (%)	1 (3.3)
ANAs, *n*/*N* (%)	25/29 (86.2)

n: number of patients positive for the variable of interest; N: number of patients without missing information regarding the variable of interest; HRCT: high-resolution computed tomography; FVC: forced vital capacity; SB DLCO: single-breath diffusing capacity of the lung for CO; DLCO-VA: diffusing capacity of the lung for CO corrected for alveolar volume; DAS: disease activity score; CK: creatine kinase; IU/L: international units per liter; Jo1: histidyl tRNA synthetase; MDA5: melanoma differentiation-associated gene 5; PL7, PL12: anti-alanyl tRNA synthetase; PM/Scl: polymyositis/scleroderma; TIF1γ: transcription intermediary factor 1-gamma; NOR90: nucleolar organizer region 90; SAE: small ubiquitin-like modifier activating enzyme; ANAs: antinuclear antibodies.

**Table 3:** Response-to-treatment analysis in patients with interstitial lung disease: variations in FVC, SB DLCO, and DLCO-VA (% predicted).

Treatment Regimens ^1^	Total (N = 31)	*p* Value *
**Mycophenolate mofetil (MMF), *n* (%)**	25 (80.6)	
∆ FVC % of predicted value (mean variation ± DP)	6.2 ± 20.9	0.160
∆ SB DLCO % (mean variation ± DP)	5.8 ± 14.2	0.058
∆ DLCO-VA % (mean variation ± DP)	4.3 ± 14.1	0.174
**Hydroxychloroquine (HCQ), *n* (%)**	19 (61.3)	
∆ FVC % (mean variation ± DP)	5.8 ± 23.2	0.291
∆ SB DLCO % (mean variation ± DP)	6.1 ± 15.1	0.103
∆ DLCO-VA % (mean variation ± DP)	2.9 ± 13.3	0.417
**Methotrexate (MTX), *n* (%)**	14 (45.2)	
∆ FVC % (mean variation ± DP)	4.4 ± 15.9	0.323
∆ SB DLCO % (mean variation ± DP)	4.5 ± 11.3	0.176
∆ DLCO-VA % (mean variation ± DP)	2.1 ± 10.2	0.471
**Azathioprine (AZA), *n* (%)**	12 (38.7)	
∆ FVC % (mean variation ± DP)	6.7 ± 23.5	0.345
∆ SB DLCO % (mean variation ± DP)	9.1 ± 12.5	**0.029**
∆ DLCO-VA % (mean variation ± DP)	9.4 ± 11.4	**0.029**
**Rituximab (RTX), *n* (%) ^2^**	11 (35.5)	
∆ FVC % (mean variation ± DP)	12.2 ± 19.9	0.084
∆ SB DLCO % (mean variation ± DP)	7.4 ± 17.4	0.211
∆ DLCO-VA % (mean variation ± DP)	−0.19 ± 12.2	0.967
**Intravenous immunoglobulin (IVIg), *n* (%)**	8 (25.8)	
∆ FVC % (mean variation ± DP)	17.0 ± 16.8	**0.025**
∆ SB DLCO % (mean variation ± DP)	14.2 ± 12.8	**0.016**
∆ DLCO-VA % (mean variation ± DP)	8.2 ± 9.5	0.086
**Cyclophosphamide (Cyc), *n* (%)**	6 (19.4)	
∆ FVC % (mean variation ± DP)	10.0 ± 18.9	0.251
∆ SB DLCO % (mean variation ± DP)	9.4 ± 18.4	0.267
∆ DLCO-VA % (mean variation ± DP)	4.6 ± 12.2	0.445
**MMF + RTX, *n* (%)**	9 (29.0)	
∆ FVC % (mean variation ± DP)	11.5 ± 22.3	0.189
∆ SB DLCO % (mean variation ± DP)	9.8 ± 18.1	0.170
∆ DLCO-VA % (mean variation ± DP)	2.3 ± 13.4	0.689
**MMF + IVIg, *n* (%)**	7 (22.6)	
∆ FVC % (mean variation ± DP)	16.6 ± 18.1	0.052
∆ SB DLCO % (mean variation ± DP)	15.2 ± 13.5	**0.025**
∆ DLCO-VA % (mean variation ± DP)	11.4 ± 6.2	**0.015**
**RTX + IVIg, *n* (%)**	6 (19.4)	
∆ FVC % (mean variation ± DP)	21.2 ± 17.1	**0.029**
∆ SB DLCO % (mean variation ± DP)	16.3 ± 14.5	**0.040**
∆ DLCO-VA % (mean variation ± DP)	4.4 ± 8.8	0.397
**RTX + MMF + IVIg, *n* (%)**	5 (16.1)	
∆ FVC % (mean variation ± DP)	21.4 ± 19.1	0.066
∆ SB DLCO % (mean variation ± DP)	18.1 ± 15.4	0.059
∆ DLCO-VA % (mean variation ± DP)	8.3 ± 5.0	0.103

^1^ Only represented treatment regimens with *n* ≥ 5. ^2^ Patients treated with RTX (*n* = 11) include 6 with antisynthetase syndrome (ASyS) and 2 with MDA5-positive dermatomyositis. ∆ Difference between baseline and the last available PFTs. * Refers to the variation in PFT values between baseline and the last visit. Values in bold indicate statistical significance at the 0.05 level (*p* < 0.05).

## Data Availability

The data underlying this article will be shared upon reasonable request to the corresponding author.
